# MED15, transforming growth factor beta 1 (TGF-β1), FcγRIII (CD16), and HNK-1 (CD57) are prognostic biomarkers of oral squamous cell carcinoma

**DOI:** 10.1038/s41598-020-65145-3

**Published:** 2020-05-21

**Authors:** Maryam Elahi, Vahid Rakhshan

**Affiliations:** 1 Department of Oral Pathology, Alborz University of Medical Sciences, Karaj, Iran; 2 Department of Cognitive Neuroscience, Institute for Cognitive Science Studies, Tehran, Iran

**Keywords:** Cancer genetics, Cancer epigenetics

## Abstract

Owing to the high incidence and mortality of oral squamous cell carcinoma (OSCC), knowledge of its diagnostic and prognostic factors is of significant value. The biomarkers ‘CD16, CD57, transforming growth factor beta 1 (TGF-β1), and MED15’ can play crucial roles in tumorigenesis, and hence might contribute to diagnosis, prognosis, and treatment. Since there was no previous study on MED15 in almost all cancers, and since the studies on diagnostic/prognostic values of the other three biomarkers were a few in OSCC (if any) and highly controversial, this study was conducted. Biomarker expressions in all OSCC tissues and their adjacent normal tissues available at the National Tumor Bank (n = 4 biomarkers × [48 cancers + 48 controls]) were estimated thrice using qRT-PCR. Diagnostic values of tumors were assessed using receiver-operator characteristic (ROC) curves. Factors contributing to patients’ survival over 10 years were assessed using multiple Cox regressions. ROC curves were used to estimate cut-off points for significant prognostic variables (α = 0.05). Areas under the curve pertaining to diagnostic values of all markers were non-significant (*P* > 0.15). Survival was associated positively with tumoral upregulation of TGF-β1 and downregulation of CD16, CD57, and MED15. It was also associated positively with younger ages, lower histological grades, milder Jacobson clinical TNM stages (and lower pathological Ns), smaller and thinner tumors, and surgery cases not treated with incisional biopsy (Cox regression, *P* < 0.05). The cut-off point for clinical stage –as the only variable with a significant area under the curve– was between the stages 2 and 3. Increased TGF-β1 and reduced CD16, CD57, and MED15 expressions in the tumor might independently favor the prognosis. Clinical TNM staging might be one of the most reliable prognostic factors, and stages above 2 can predict a considerably poorer prognosis.

## Introduction

Oral squamous cell carcinoma (OSCC) is a common oral cancer (90% of oral cancers) and has a poor prognosis^1–4^. It is aggressive and can modulate the immune system through evasion and direct/indirect suppression^3,5–7^. High rates of recurrence despite numerous treatments imply that current treatments and prognostic predictors are not efficient^3,7,8^. These call for investigating new diagnostic, prognostic, and possibly therapeutic markers for SCC. Many factors might play a role in cancer prognostication, including tobacco, alcohol, human papilloma virus, demographic/clinical/histopathological factors (such as stage, grade, or tumor budding), and biomarkers (such as Gas6)^9–11^. SCCs of head and neck might be immune-modulatory, and the prognostic effects of immune system activity have been conflicting^3,5,12–17^. The interaction between tumor cells and the immune system is crucial in tumorigenesis and improved knowledge of dysregulated pathways might allow identification of new targets^18,19^. Therefore, the assessment of diagnostic and prognostic roles of biomarkers such as transforming growth factor beta 1 (TGF-β1), CD16, CD57, and MED15 is of significant value.

Low-affinity immunoglobulin gamma Fc region receptor III (FcγRIII, CD16) is an IgG Fc receptor found on the surface of inflammatory cells^20^. One of the main routes of defense against tumor cells (and viral/bacterial infections) is antibody-dependent cellular cytotoxicity (ADCC) in which, immunoglobulin G antibody attaches to the target cell; the Fc domains of these antibodies then bind to Fc receptors expressed on leukocytes (such as natural killer [NK] cells), triggering the release of cytotoxic granules or upregulating the expression of death receptors on the surface of the target cell^18,21–25^. Therefore, it is anticipated to increase in tumoral tissues, and therefore probably act as a diagnostic and prognostic factor. Human studies on the prognostic role of CD16 in different cancers are a few and controversial^18^. In OSCC, it has not been assessed in humans except a recent immunohistochemistry survival analysis (which has studied NK cells only and has not evaluated any factors associated with its tumoral expression)^18^. To the best of our knowledge, its role as a diagnostic marker of OSCC has not been established either.

Mature and differentiated NK cells also express CD57 which makes them more cytotoxic and reactive to signaling via CD16^18,26^. CD57 (HNK-1, Leu 7) is a sulfated carbohydrate chain surface antigen containing the epitope for the antibody HNK-1, usually expressed in T-lymphocytes and NK cells^18,26–29^. Although its role is not yet completely understood^29^, heterogeneous patterns of increased and decreased expressions of CD57 have been observed in cancer^29,30^. Despite the importance of the interaction of immune system and tumor cells, the expression of CD57 and development/prognosis of head and neck SCC have not been adequately studied in human^28,31^. Additionally, the results have been controversial marking negative and positive^28,32^ associations between CD57 upregulation and survival. Very few studies have assessed its diagnostic role in few cancers^28,29,33–35^.

Transforming growth factor beta 1 (TGF-β1) is a pleiotropic cytokine with diverse and paradoxical effects^7,36–39^. It can suppress tumorigenesis via its fibrogenic and antiproliferative effects, reducing metastasis likelihood^40,41^. Also it might contribute to malignancy, tumor angiogenesis, tissue invasion, metastasis, and neoplastic transformation of epithelial cells, immune suppression, and epithelial hyperproliferation^7,36,38,41–44^. The role of TGF-β1 in SCC prognosis and diagnosis has been controversial^7^ and still needs further research. Disruption of TGF-β1 signaling pathways might be approached to control the tumorigenesis^41^, although some authors do not find it a useful prognostic factor^7,45^. Since it has extremely paradoxical effects even in one type of cancer depending on the stage and severity of the cancer (let alone in different cancers), and since the results have been quite controversial, the examination of its diagnostic and prognostic roles is of clinical and scientific value.

MED15 is a subunit of the tail module of the mediator multiprotein complex and is a key regulator of TGF-β signal transduction^19,46,47^. Mediator is a main regulator of protein coding-genes, and an integrative hub for numerous signaling pathways^47,48^. Mediator subunits have been recently suggested to be linked to cancer (plus metabolic, cardiovascular, and neurological disorders) but this is a very new topic and needs more evaluation^47,48^. Despite its importance for regulating TGF-β signaling (which plays crucial roles in SCC^19^), prognostic role of MED15 is not assessed except in a few recent studies on prostate and head and neck cancers^19,48,49^. Besides, its diagnostic roles remain unaddressed.

This study was conducted since (1) MED15 is not evaluated in any cancers except partially in very few recent studies on certain cancers, (2) reports on the other three biomarkers are controversial, non-existent (in the case of diagnostic roles), or scarce (in the case of head and neck SCCs), (3) no studies have assessed these biomarkers together, and therefore their effects on the survival have not been evaluated when controlling for the other ones; and (4) many previous studies on these cancer biomarkers have used less accurate methods such as IHC and have examined fewer markers (mostly limited to one or two). Research goals were (A) to assess the diagnostic role of each of these markers, (B) to determine their prognostic role by investigating the influences of these markers (as well as other clinicopathological factors) on patients’ 123-month survival, (C) to determine cut-off points for the identified prognostic variables, and (D) to estimate the prognostic role of other clinicopathological factors.

## Materials and methods

### Tissue samples

This retrospective case-control study was performed on 48 tumoral tissues and 48 genetically-matched adjacent healthy tissues as controls. The sample size was determined as All the OSCC specimens available at the National Tumor Bank (n = 384 biomarker data points = 4 markers × [48 OSCC cancer tissues + 48 control tissues]). Biological materials were provided by the National Tumor Bank which is founded by the Cancer Institute for Cancer Research. As the eligibility criteria, all patients with oral SCC who underwent surgery in the Institute were selected for this study. None of the selected patients had received any chemotherapy or radiotherapy prior to surgery. The patients were diagnosed with OSCC based on histopathological examinations at two time points by at least two pathologists. Patients’ pathological records (including the histology grade, tumor size, and Jacobson clinical TNM [tumor, node, and metastasis]) staging were recorded. Subjects with chronic or acute inflammatory diseases or any other synchronized primary tumor were also excluded from the study. The protocol ethics were approved by the Research Committee of the Alborz University of Medical Sciences, Karaj, Iran (approved as theses 1395–113 and 1395–118). All specimens were prepared with full observation of preparation and preservation processes of standard protocols in accordance with ethical permissions. Ethics of the study were approved by the research committee of the university, and written informed consents had been obtained from all patients. The data were checked for consistency and correctness for numerous times. Tumor measurements were re-performed twice by two different pathologists on all the 48 paraffin-embedded specimens to ensure a high accuracy of the recorded data.

At the end of the 10-year study period (ending in 2017), 28 out of 48 patients were deceased. The average duration of survival since the diagnosis was 33.4 ± 35.5 months in the whole sample (minimum: 1 week, Q1: 4.5 months, median: 23.5 months, Q3: 45.5 months, maximum:123 months [i.e., the follow-up period]). Among the deceased patients, the mean survival duration was 13.7 ± 22.5 months (minimum: 1 week, Q1: 5 weeks, median:9 months, Q3:15 months, maximum:115 months).

Patients had been diagnoses with SCC between 2007 and 2015. The mean follow-up duration (from diagnosis to death or from diagnosis to the final follow-up) was 33.4 ± 35.5 months (minimum: 1 week, Q1: 4 months, median: 23 months, Q3: 45 months, maximum: 123 months). Of them, 29 were males and 19 were females. Their average age at diagnosis was 63.8 ± 15.3 years . Family history of previous cancers existed in 10 patients. Only one patient disclosed alcohol drinking. Only 7 were cigarette smokers at the time of diagnosis.

Continuous variables of the tumors are presented in Table 1. At the end of the follow-up duration, 28 patients had deceased and 20 were alive. Of tumors, 16 were in the labial mucosa and buccal mucosa, 16 were in the tongue, 7 were in the mouth floor, and the rest were in the lower gingiva (3), oropharynx (1), and not specified (or multisite OSCC) (4). Histology grades were I, II, and III in 28, 16, and 3 patients. Necrosis was present in 10 patients. Lymphatic invasion was present in 10 cases. Vascular invasion was seen in 9 cases. Perineural invasion was positive in 18 patients. Extracapsular nodal extension was present in 3 patients. Pathological T modes were T1, T2, T3, and T4 in 6, 13, 12, 16 patients, respectively. Pathological N modes were N0, N1, and N2 in 32, 4, and 11 cases, respectively. Clinical metastases were M0 in 46 cases and M1 in one case. The stages 1, 2, 3, and 4 were seen respectively in 4, 6, 11, and 26 patients. One, one, and 45 patients had undergone fine needle biopsy, incisional biopsy, and excisional biopsy, respectively.

**Table 1:** Descriptive statistics of continuous variables including demographics, tumor characteristics, ΔCts, and ΔΔCts.

Variable	N	Mean	SD	95% CI		Min	Q1	Med	Q3	Max
Patient Age	48	63.81	15.33	59.36	68.26	23.37	57.91	64.7	75.74	90.39
Tumor Size	47	47.13	25.81	39.55	54.7	15	30	40	70	120
Tumor Volume	47	58.93	135.8	19.06	98.8	0.75	8.4	14	56.87	864
Tumor Depth	46	18.18	14.96	13.74	22.63	1.5	7.0	18.5	25.0	80.0
ΔCt CD16 tumoral	48	−3.841	3.054	−4.728	−2.954	−10.38	−6.034	−4.09	−1.611	2.78
ΔCt CD16 normal	48	−3.666	3.023	−4.544	−2.788	−11.49	−5.829	−3.215	−1.735	1.78
ΔΔCt CD16	48	−0.1752	4.176	−1.388	1.037	−9.19	−3.395	−0.11	2.613	9.88
ΔCt CD57 tumoral	48	−6.05	3.808	−7.156	−4.944	−12.5	−8.845	−5.633	−3.738	3.115
ΔCt CD57 normal	48	−5.269	3.408	−6.258	−4.279	−14.35	−6.894	−4.617	−2.922	−0.215
ΔΔCt CD57	48	−0.7829	3.769	−1.877	0.3114	−8.16	−3.458	−1.055	1.385	7.83
ΔCt TGF-β1 tumoral	48	−4.187	3.376	−5.168	−3.207	−11.01	−6.045	−4.038	−1.97	4.43
ΔCt TGF-β1 normal	48	−4.148	3.346	−5.119	−3.176	−12.62	−5.915	−3.663	−1.705	4.143
ΔΔCt TGF-Β1	48	−0.0394	4.135	−1.24	1.161	−9.69	−2.258	−0.58	2.495	9.93
ΔCt MED15 tumoral	48	−4.426	3.324	−5.392	−3.461	−10.68	−6.19	−4.118	−2.803	4.208
ΔCt MED15 normal	48	−3.844	3.866	−4.967	−2.722	−13.48	−6.403	−3.743	−1.29	3.155
ΔΔCt MED15	48	−0.5825	3.422	−1.576	0.4112	−8.2	−2.64	−0.475	1.49	8.49

SD, standard deviation; CI, confidence interval; Min, minimum; Q1, first quantile; Med, median; Q3, third quantile; Max, maximum.

### RNA extraction and real-time quantitative polymerase chain reaction (qRT-PCR)

The qRT-PCR procedure was performed thrice for each of the 96 cancerous and benign tissues. Primer sequences were synthesized for TGF-β1 (left: AGCTGTACATTGACTTCCGC, right: GTCCAGGCTCCAAATGTAGG), MED15 (left: AGAACTTCAGTGTCCCCTCA, right: GTACTTCGACAGCTGCTTCA), CD16 (left: GTGGGTGTTCAAGGAGGAAG, right: CTGCCTTTGCCATTCTGTAA), and CD57 (left: GAACTTGTCACCCTCAACGA, right: CTTCTTGCCCTCATTCACC). The RNA was extracted using a Qiagen kit (Germantown, USA) according to the manufacturer’s instructions. After normalization of all the extracted RNAs to 1 µg, the RNA was reverse-transcribed into single-strand cDNA using a Thermo kit (Thermo Fisher Scientific, Waltham, Massachusetts, USA). The quantity and purity of extracted RNA was analyzed using Nano-Drop Technologies (ND-2000). The product was used for quantitative qRT-PCR using SYBR green/ROX (Takara, Japan) real-time PCR master mix according to the protocol of Bioneer RT-PCR thermal cycler. The amplification protocol comprised 1 cycle at 95 °C for 4 min followed by 40 cycles at 95 °C for 15 s, 60 °C for 30 s, and then 72 °C for 30 s. The relative expression of the studied genes to the housekeeping gene (β-actin) was calculated by measuring the Delta threshold cycle value (ΔCt) for each sample (i.e., Ct_[Housekeeping]_ − Ct_[Target]_). The Delta Delta cycle value (ΔΔCt) as the +log_2_-fold-change was then calculated from the difference between the ΔCt of the tumoral tissue and the ΔCt of its normal adjacent tissue (i.e., tumor ΔCt – the ΔCt of its adjacent benign tissue). The fold-change (ratio) in the expression of the target gene in the tumoral tissue to its expression in the healthy tissue was then calculated by the 2^ΔΔCt^ formula^50^. This way, a ΔΔCt above zero would indicate a logarithmic increase in the expression of the marker in the tumoral tissue compared with its adjacent tissue. Also a fold-change value above 1 would point to a tumoral overexpression compared with the control tissue.

### Statistical analysis

As expression indices and log-fold-change values, ΔCts and ΔΔCts were used for analyses. According to the D’Agostino & Pearson omnibus normality test, the ΔΔCt values were normally distributed. Descriptive statistics as well as 95% confidence intervals (CI) were calculated for all continuous variables. Tumoral ΔCt values were compared with control ΔCt values using a paired-samples *t*-test, in order to assess if the average gene expression in the tumor was different from the average normal tissue gene expression. Correlations between ΔCt values were assessed using a Pearson coefficient. A receiver-operator characteristic (ROC) curve was used to estimate the diagnostic accuracy of ΔCts in discriminating tumoral tissues from healthy ones. A multiple Cox regression was used to assess the prognostic role of each of the biomarkers when other factors were controlled for. In order to select proper models, items such as model estimates, multiple imputation results, principal component analysis results, model significance, variance inflation factors, and the number of significant results per model were taken into account. When important variables caused multicollinearity, they would be modeled interchangeably in rather similar but separate models. A ROC curve was used to identify potential cut-off points for death prediction, through evaluating prognostic sensitivity and specificity of the variables turned significant in the Cox models. A Kaplan-Meier function was used for drawing the cumulative survival curves of dichotomized variables. The level of significance was set at 0.05.

## Results

### Diagnostic factors

**Figure 1:**
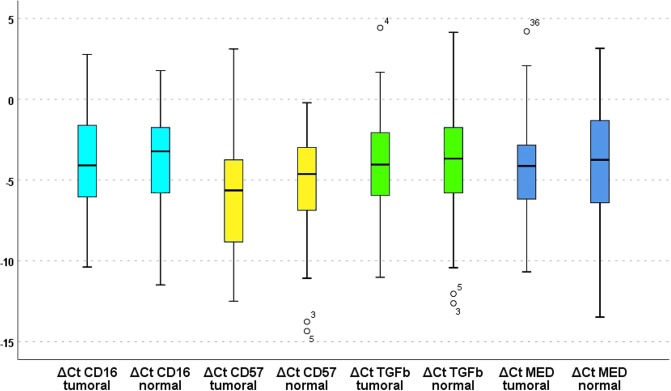
Boxplots presenting medians, quartiles, minima, and maxima for ΔCt of the four biomarkers in tumoral and benign tissues.

The paired-samples *t*-test did not detect significant differences between tumoral versus control ΔCt values of CD16 (*P* = 0.772), CD57 (*P* = 0.157), TGF-β1 (*P* = 0.947), and MED15 (*P* = 0.244): None of the relative expressions of the four evaluated genes leaned towards an overall overexpression or underexpression in the tumor compared to the adjacent healthy tissue (Fig. 1, Table 1). There was not a significant correlation between tumoral and normal ΔCt values for CD16 or TGF-β1, but the correlations between tumoral and normal ΔCt values of CD57 and MED15 were significant (Table 2).

**Table 2:** The Pearson correlation matrix between ΔCt values, indicating significant positive correlations between cancerous and normal tissues as well as significant correlations among different biomarkers.

		ΔCt CD16 tumoral	ΔCt CD16 normal	ΔCt CD57 tumoral	ΔCt CD57 normal	ΔCt TGF-β1 tumoral	ΔCt TGF-β1 normal	ΔCt MED15 tumoral
ΔCt CD16 normal	R	**0.056**						
	*P*	**0.704**						
ΔCt CD57 tumoral	R	**0.741**	0.203					
	*P*	**0.000**	0.166					
ΔCt CD57 normal	R	0.267	**0.749**	**0.460**				
	*P*	0.066	**0.000**	**0.001**				
ΔCt TGF-β1 tumoral	R	**0.545**	0.001	**0.426**	0.195			
	*P*	**0.000**	0.997	**0.003**	0.184			
ΔCt TGF-β1 normal	R	0.050	**0.707**	0.106	**0.580**	0.244		
	*P*	0.734	**0.000**	0.474	**0.000**	0.095		
ΔCt MED15 tumoral	R	**0.686**	**0.337**	**0.542**	**0.361**	**0.475**	**0.352**	
	*P*	**0.000**	**0.019**	**0.000**	**0.012**	**0.001**	**0.014**	
ΔCt MED15 normal	R	0.269	**0.698**	0.224	**0.546**	0.204	**0.699**	**0.556**
	*P*	0.064	**0.000**	0.125	**0.000**	0.163	**0.000**	**0.000**

**Figure 2:**
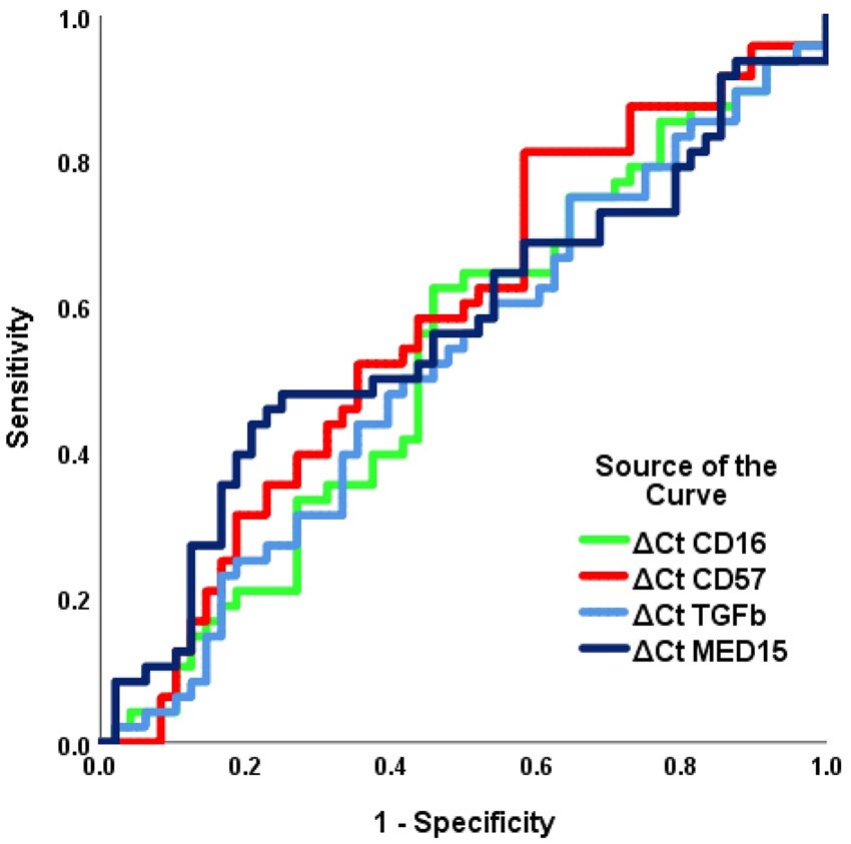
ROC curves computed based on sensitivity and specificity of cancer determination (from normal tissue) using ΔCt values.

The ROC curve did not identify significant areas under the curve (AUC) for differentiating tumoral tissues from normal controls, based on ΔCt values of CD16 (AUC [SE] = 0.530 ± 0.060, 95% CI = 0.413–0.646, *P* = 0.618), CD57 (AUC [SE] = 0.578 ± 0.059, 95% CI = 0.462–0.693, *P* = 0.190), TGF-β1 (AUC [SE] = 0.521 ± 0.059, 95% CI = 0.404–0.637, *P* = 0.725), and MED15 (AUC [SE] = 0.567 ± 0.059, 95% CI = 0.450–0.683, *P* = 0.259, Fig. 2).

### Prognostic factors affecting the survival

Since some of variables were considerably correlated, they were first detected by evaluating correlation matrixes, VIFs, results of multiple-imputation, and results of a component factor analysis. After detecting bivariate correlations (between independent factors and survival duration and the live/deceased status) and potentially significant variables, two Cox regression models were conducted, each with a group of the least correlated variables. Tumoral upregulation of CD16, CD57, and MED15 were associated with increased risk of mortality, while overexpression of TGF-β1 might improve prognosis. Older ages, higher stages/grades, higher Ns, as well as larger tumor volumes might deteriorate the prognosis. Incisional biopsy might as well be associated with increased mortality (Tables 3 to 5). Depth of invasion was as well associated with mortality (Table 5).

**Table 3:** Factors (including the biomarkers) contributing to the survival of SCC patients, computed using the Cox regression.

Original model −2 Log Likelihood = 150.585	B	SE	Wald	*P*	HR	95% CI for HR	
Sex: Male	0.378	0.578	0.428	0.513	1.459	0.470	4.527
Age at Diagnosis	0.042	0.023	3.182	0.074	1.042	0.996	1.091
Tumor Volume (ml)	0.003	0.002	3.940	0.047	1.003	1.000	1.006
Histology Grade	0.845	0.439	3.700	0.054	2.329	0.984	5.511
Necrosis Presence	0.435	0.597	0.530	0.467	1.545	0.479	4.979
Lymphatic Invasion	−0.065	1.186	0.003	0.956	0.937	0.092	9.584
Vascular invasion	0.321	1.168	0.076	0.783	1.378	0.140	13.590
Perineural invasion	−0.084	0.593	0.020	0.887	0.919	0.287	2.942
Extracapsular nodal extension	−0.283	1.039	0.074	0.785	0.754	0.098	5.774
Stage	0.831	0.368	5.113	**0.024**	2.296	1.117	4.718
Smoking	0.365	0.806	0.206	0.650	1.441	0.297	6.992
Site of primary	0.131	0.123	1.141	0.286	1.140	0.896	1.451
Type of procedure (reference)			7.397	**0.025**			
Type of procedure (excisional biopsy)	−9.988	501.660	0.000	0.984	0.000	0.000	
Type of procedure (incisional biopsy)	5.462	2.008	7.397	**0.007**	235.475	4.598	12059
Family History	−0.243	0.594	0.168	0.682	0.784	0.245	2.511
ΔΔCt CD16	0.354	0.140	6.359	**0.012**	1.425	1.082	1.877
ΔΔCt TGF-β1	−0.560	0.170	10.834	**0.001**	0.571	0.409	0.797
ΔΔCt MED15	0.275	0.144	3.640	0.056	1.317	0.993	1.746
**Optimized model −2 Log Likelihood** = **151.842**							
Sex: Male	0.580	0.523	1.228	0.268	1.786	0.640	4.979
Age at Diagnosis	0.047	0.019	6.044	**0.014**	1.048	1.010	1.089
Tumor Volume (ml)	0.003	0.001	4.610	**0.032**	1.003	1.000	1.006
Histology Grade	0.908	0.404	5.035	**0.025**	2.478	1.122	5.475
Stage	0.724	0.318	5.182	**0.023**	2.062	1.106	3.847
Site of primary	0.142	0.112	1.592	0.207	1.152	0.925	1.435
Type of procedure (reference)			11.545	**0.003**			
Type of procedure (excisional biopsy)	−9.752	510.340	0.000	0.985	0.000	0.000	
Type of procedure (incisional biopsy)	4.936	1.453	11.545	**0.001**	139.226	8.075	2401
ΔΔCt CD16	0.337	0.137	6.035	**0.014**	1.401	1.071	1.834
ΔΔCt TGF-β1	−0.515	0.144	12.717	**0.000**	0.598	0.450	0.793
ΔΔCt MED15	0.285	0.118	5.796	**0.016**	1.330	1.054	1.677

The variables age, tumor volume, grade, stage, procedure type, ΔΔCt CD16, ΔΔCt TGF-β1, and ΔΔCt MED15 became significant in the optimized model.

B, regression coefficient; SE, standard error; HR, hazard ratio; CI, confidence interval.

**Table 4:** Factors contributing to the survival of SCC patients (including the biomarkers), computed using the Cox regression.

Original model −2 Log Likelihood = 154.780	B	SE	Wald	*P*	HR	95% CI for HR	
Sex: Male	0.281	0.620	0.206	0.650	1.325	0.393	4.470
Age at Diagnosis	0.036	0.020	3.175	0.075	1.037	0.996	1.079
Tumor Volume (ml)	0.003	0.002	3.930	**0.047**	1.003	1.000	1.006
Histology Grade	0.858	0.452	3.593	0.058	2.358	0.971	5.723
Necrosis Presence	0.391	0.532	0.541	0.462	1.479	0.522	4.191
Vascular invasion	−0.008	0.611	0.000	0.990	0.992	0.300	3.285
Perineural invasion	−0.095	0.562	0.028	0.866	0.910	0.302	2.736
Extracapsular nodal extension	−0.261	0.965	0.073	0.786	0.770	0.116	5.101
Stage	0.744	0.392	3.616	0.057	2.105	0.977	4.535
Smoking	−0.053	0.751	0.005	0.944	0.948	0.217	4.135
Site of primary	0.071	0.112	0.399	0.528	1.074	0.861	1.338
Type of procedure (reference)			8.547	**0.014**			
Type of procedure (excisional biopsy)	−9.211	497.527	0.000	0.985	0.000	0.000	
Type of procedure (incisional biopsy)	4.408	1.508	8.547	**0.003**	82.133	4.276	1578
Family History	0.220	0.588	0.140	0.708	1.246	0.394	3.941
ΔΔCt CD57	0.211	0.127	2.746	0.098	1.234	0.962	1.583
ΔΔCt TGF-β1	−0.301	0.114	7.009	**0.008**	0.740	0.593	0.925
ΔΔCt MED15	0.204	0.146	1.950	0.163	1.226	0.921	1.633
**Optimized model −2 Log Likelihood** = **155.083**							
Age at Diagnosis	0.040	0.018	4.911	**0.027**	1.040	1.005	1.077
Tumor Volume (ml)	0.003	0.001	3.986	**0.046**	1.003	1.000	1.006
Histology Grade	0.850	0.429	3.931	**0.047**	2.341	1.010	5.426
Necrosis Presence	0.436	0.489	0.796	0.372	1.547	0.593	4.032
Stage	0.743	0.374	3.949	**0.047**	2.101	1.010	4.372
Site of primary	0.070	0.104	0.453	0.501	1.072	0.875	1.315
Type of procedure (reference)			9.658	**0.008**			
Type of procedure (excisional biopsy)	−9.311	491.243	0.000	0.985	0.000	0.000	
Type of procedure (incisional biopsy)	4.392	1.413	9.658	**0.002**	80.807	5.064	1290
Family History	0.233	0.561	0.173	0.678	1.263	0.420	3.794
ΔΔCt CD57	0.237	0.107	4.936	**0.026**	1.267	1.028	1.562
ΔΔCt TGF-β1	−0.292	0.097	9.009	**0.003**	0.747	0.617	0.904
ΔΔCt MED15	0.189	0.114	2.752	0.097	1.207	0.966	1.509

The variables age, tumor volume, grade, stage, procedure type, ΔΔCt CD57, and ΔΔCt TGF-β1 became significant in the optimized model, while ΔΔCt MED15 became marginally significant.

B, regression coefficient; SE, standard error; HR, hazard ratio; CI, confidence interval.

**Table 5:** Factors contributing to the survival of SCC patients (including the biomarkers), computed using the Cox regression.

Optimized model-2 Log Likelihood = 151.374	B	SE	Wald	*P*	HR	95% CI for HR	
Age at Diagnosis	0.044	0.019	5.439	**0.020**	1.045	1.007	1.084
Depth of Invasion (mm)	0.033	0.016	4.289	**0.038**	1.033	1.002	1.065
Lymphatic Invasion	0.712	0.608	1.370	0.242	2.037	0.619	6.708
Pathological N	0.657	0.264	6.179	**0.013**	1.929	1.149	3.238
Site of primary	0.146	0.115	1.611	0.204	1.157	0.924	1.449
Type of procedure (reference)			7.491	**0.024**			
Type of procedure (excisional biopsy)	−11.889	499.990	0.001	0.981	0.000	0.000	
Type of procedure (incisional biopsy)	3.694	1.350	7.490	**0.006**	40.215	2.854	566.697
Family History	−0.556	0.596	0.869	0.351	0.574	0.178	1.845
ΔΔCt CD16	0.279	0.111	6.297	**0.012**	1.321	1.063	1.642
ΔΔCt TGF-β1	−0.324	0.112	8.432	**0.004**	0.723	0.581	0.900
ΔΔCt MED15	0.128	0.078	2.702	0.100	1.136	0.976	1.323

The variables age, depth of invasion, pathological N, procedure type, ΔΔCt CD16, and ΔΔCt TGF-β1 became significant, while ΔΔCt MED15 became marginally significant.

B, regression coefficient; SE, standard error; HR, hazard ratio; CI, confidence interval.

### Cut-off points for death prediction

**Figure 3:**
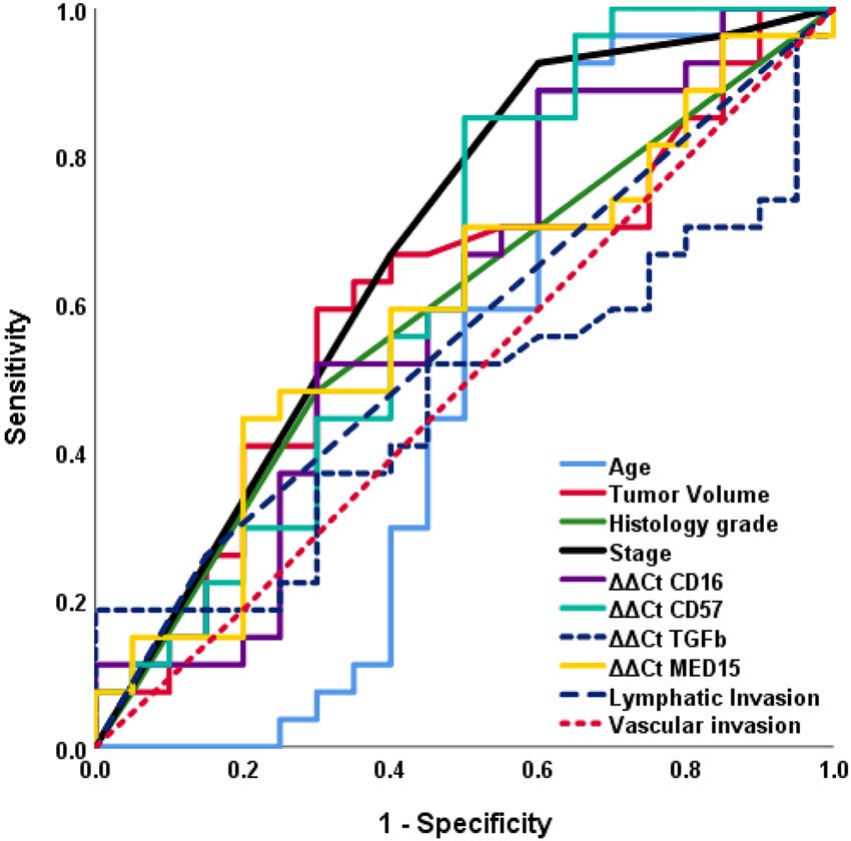
ROC curves of the variables contributing to the survival.

A ROC curve was used to identify which of the factors contributing to the survival can be useful for mortality prediction. The only variable with an area under the curve significantly differing from 50% was ‘stage’ (Fig. 3, Table 6). The cut-off point of the variable ‘stage’ for death prediction (as the stage yielding the greatest sum of sensitivity and specificity) was determined as between the stages 2 and 3 (sensitivity = 0.926, specificity = 0.600).

**Table 6:** Areas under the ROC curves of the variables contributing to survival, indicating the significance of the variable stage.

Variables	Area	SE	Asymptotic *P*	Asymptotic 95% CI	
Patient Age	0.493	0.098	0.931	0.300	0.685
Tumor Volume	0.602	0.085	0.237	0.435	0.768
Tumor Histology Grade	0.590	0.084	0.297	0.425	0.755
Tumor Stage	0.676	0.082	**0.041**	0.514	0.837
ΔΔCt CD16	0.598	0.087	0.254	0.427	0.769
ΔΔCt CD57	0.635	0.087	0.116	0.465	0.805
ΔΔCt TGF-β1	0.469	0.085	0.715	0.302	0.635
ΔΔCt MED15	0.587	0.085	0.312	0.420	0.754
Lymphatic Invasion	0.555	0.085	0.526	0.389	0.721
Vascular invasion	0.493	0.086	0.931	0.324	0.661

SE, standard error; CI, confidence interval.

### Survival plots

**Figure 4:**
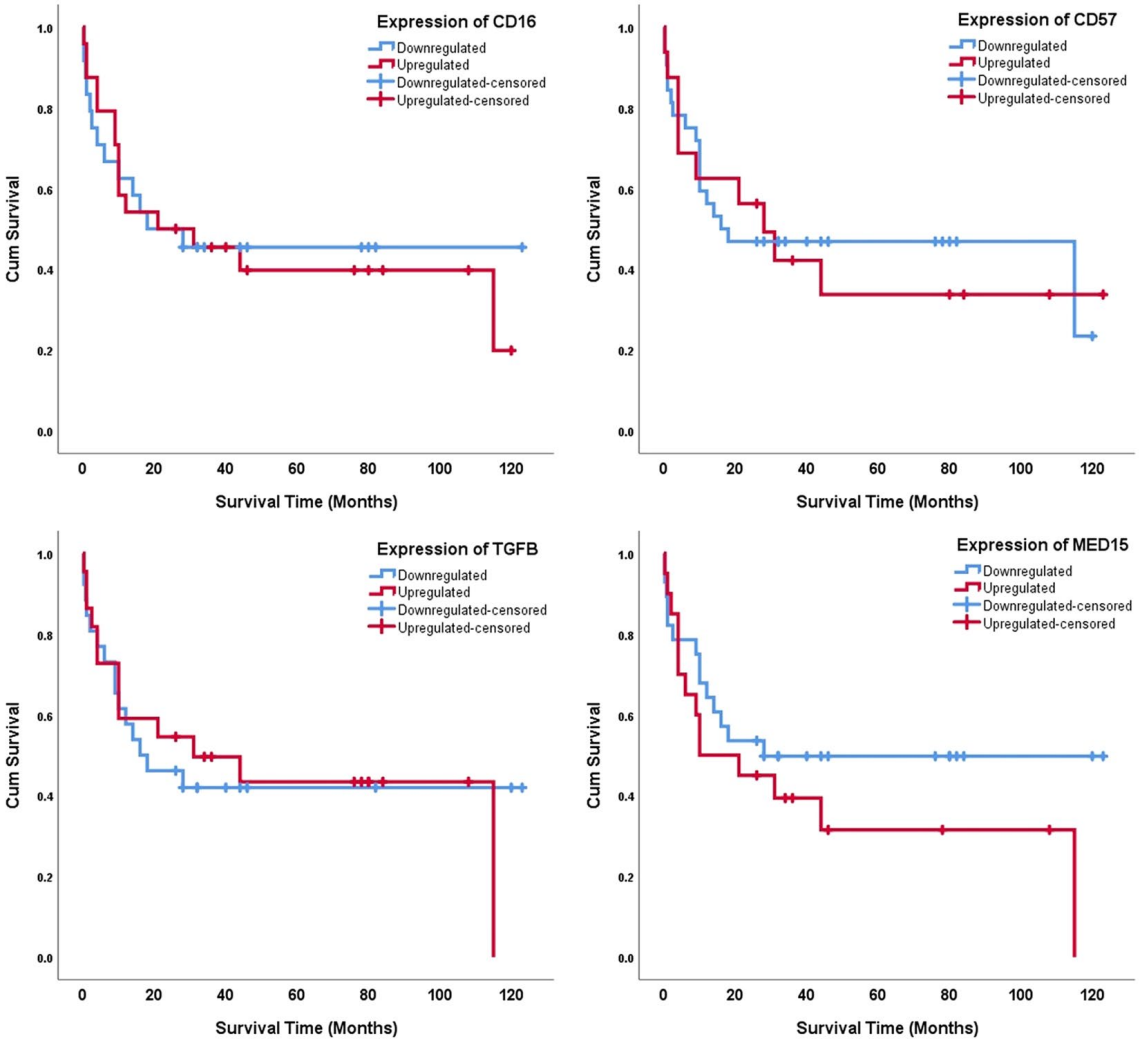
Cumulative survival plots for biomarker expressions, drawn using the Kaplan-Meier function.

**Figure 5:**
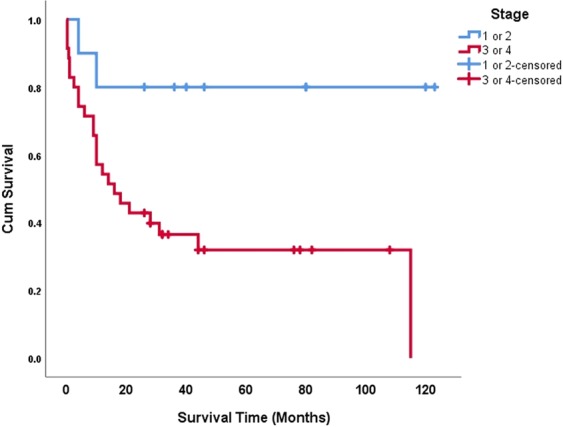
Cumulative survival plots for the TNM stage dichotomized into mild and severe stages.

Gene expressions were dichotomized into upregulation (i.e., foldchanges above 1 [or ΔΔCt above 0]) and downregulation (foldchanges below 1). The Kaplan-Meier function was used to draw cumulative survival curves for the biomarkers (Fig. 4). The variable ‘clinical TNM stage’ was as well dichotomized into two modes of mild (stages 1 and 2) and severe (stages 3 and 4); its survival plot was drawn using the Kaplan-Meier estimator (Fig. 5).

## Discussion

The findings of this study indicated that all the average expressions of biomarkers in the tumor were not different from their expressions in the adjacent benign tissue. Older ages, greater stages, relative tumoral upregulation of CD16, CD57, and MED15, as well as downregulation of TGF-β1 (compared to the expressions in the adjacent normal tissue), poorer histologic grades, and increases in tumor volume might predict a higher rate of mortality. In addition, it was found that cases undergone incisional biopsy might have a higher chance of mortality compared to those undergone surgical resection.

### Clinicopathological predictors of survival

In this study, it was observed that an increased tumor volume could increase the risk of mortality. Few essays have mentioned tumor volume, and it should be calculated using 3D imaging systems^9,51^. Tumor volume is a function of its diameter and depth. Depth of invasion has been suggested as a main factor in prognosis of different cancers^52–57^ including SCCs^57,58^. It can predict recurrence, metastasis, and death^9,59^. In this study as well, this variable acted as a predictor of survival. Each unit of increase in TNM staging was found to be the most prominent prognostic factor for survival.

Various studies have indicated that factors including regional lymph node metastasis^60–64^, lymphatic invasion^65^, histological grading^60,61,64^, the anatomic site^60^, clinical TNM staging^60,61,65,66^ and depth of invasion^60,62,63^ might be key prognostic markers of OSCC^60^ or other cancers^65^. TNM staging has been recognized as a very important prognostic factor^60,67–69^. In line with our findings, a recent study^34^ identified both the variables staging and pathological N, but they did not indicate significant roles for age or smoking^34^. Associations were found in this sample between mortality with histologic grade in this sample. Some studies have estimated an increased mortality in cases with perineural and vascular invasions^52,70–72^; we could not find such roles for these variables, possibly because of methodological and sample differences. For instance, smoking, vascular invasion, or perineural invasion were themselves correlated with tumoral overexpressions of some markers, which could mask their role in multivariable models including those markers.

Although in this study, the site of cancer was not a prognostic factor, the method of treatment was. This might imply the higher efficacy of surgical resection. In this research, the expression of all the biomarkers were positively associated with each other. Almost no other studies have assessed this.

### Diagnostic roles of biomarkers

Findings of this study indicated no significant overall difference between the expressions of either marker in the tumor with their expression in the benign adjacent tissue. The ROC curve as well did not point to significant diagnostic merits for any of these markers. Our findings were in line with the study of Wangerin *et al*.^29^ who did not recognize CD57 as a proper diagnostic marker for prostate cancer. According to them, this marker might not be specific to tumoral tissue but benign prostatic tissue as well^29^. A study on breast cancer identified CD14^+^–CD16^+^ monocytes as a proper diagnostic marker (AUC = 80.5%)^33^. In this study, we could not find any significant diagnostic role for TGF-β1. This was in contrast to studies showing significant diagnostic roles for this marker in different cancers (except SCC for which its diagnostic value had not been assessed)^73,74^ or new relevant markers such as Latent Transforming Growth Factor β Binding Protein^75^. Diagnostic role of MED15 has not been assessed before. The reason for disputes might be that cells expressing these markers are involved in numerous inflammatory responses, the number and complexity of which might mask their role as expected binary diagnostic factors.

### Prognostic values

CD16 enables NK cells to recognize and kill target cells opsonized with antibodies through ADCC^26,32^. Also CD16+ monocytes are pro-inflammatory and a major source of TNF^76^, and their numbers are increased during infection and inflammation^77–80^. A positive association was observed between the overexpression of CD16 and mortality. Few human studies exist on this subject^18^. Taghavi *et al*.^18^ could not detect a link between survival and CD16 expression in OSCC. Similarly, Lazaris *et al*.^81^ reported a lack of significant prognostic role for CD16 in laryngeal carcinoma. Grimm *et al*.^82^ as well did not find a significant association between survival and peripheral CD16 monocytes; they also did not detect different extents of such cells in tumoral and control cells^82^. Russell *et al*.^3^ as well did not find a significant prognostic role for CD16 marker. On the other hand, results of Gonzalez *et al*.^83^ indicated that in laryngeal carcinoma patients, CD16 in peripheral blood mononuclear cells correlated with nodal metastases, suggesting it as a prognostic marker^83^. Sorskaar *et al*.^84^ found an improved prognosis of lymphoblastic leukemia with increases in CD16 cells in bone marrow. Also Millrud *et al*.^85^ reported a positive correlation between increased CD16 neutrophils in peripheral blood and a better survival. Valenzuela-Membrives *et al*.^86^ observed CD16 NK cells in peripheral blood as well as normal tissue; however, CD16 NK cells were diminished in tumor stroma (although they did not assess survival prognosis)^86^. Sconocchia *et al*.^87^ as well observed a significant correlation between high CD16+ cell infiltrate with long-term survival in patients with colorectal carcinoma while they detected no prognostic roles for NK cells. *In vitro* studies have shown reduced NK killing potential and cancer immune evasion accompanied by downregulation of CD16^24,88^. The dispute might be attributed to assessment of different cancers (which differ in tumor progression mechanisms, immune response/infiltration, and immune-tumor interaction^86^), using different methods (qPCR versus IHC, or for example some studies evaluated only certain types of cells^86^), and characteristics of samples in terms of demographics and cancer severities which again can affect immune response^86^. Moreover, it is possible for cancers to evade the immune response while evoking it simultaneously^89^. Therefore, perhaps in this study, the increase in CD16 expression in more severe cases was to compensate the immunosurveillance evasion mechanisms of cancer. Moreover, it is possible that severer cancers (which had higher CD16 expressions) received more aggressive treatments, improving their survival. This study found no significant difference in expression of CD16 in tumoral cells versus benign adjacent tissues; however, according to some authors, CD16 is downregulated in HNSCC^90^.

Patient survival was associated negatively with CD57 upregulation in the tumor compared to benign adjacent tissue. This was similar to results of studies on OSCC^34^, renal cell carcinoma^91^, melanoma^92^, gastric carcinoma^65^, multiple myeloma^93^, lymphoma and leukemia^84,94,95^. Nonetheless, our finding was in contrast to the results of other studies which found better survivals in patients having a higher level of tumoral CD57 NK cells in head and neck SCC, lung SCC, breast carcinoma, esophageal carcinoma, metastatic carcinoma, gastric carcinoma, and colorectal cancer^18,28,32,66,96–99^. Karpathiou *et al*.^100^ reported that increases in CD57 T cells predict a better response to chemotherapy, reduced metastasis, and better prognoses. This association might be due to various factors such as diminished MHC Class I expression in some tumors which disallow T-lymphocytes immunosurveillance and make the role of NK cells more prominent^32,101,102^. Adachi *et al*.^103^ observed that in early stages of colorectal cancer, CD57 NK cells might increase in the lymph nodes but they might not infiltrate into the tumor; this nodal increase might positively predict survival^103^. Similarly, Hermann *et al*.^104^ found reduced anticancer cytotoxicity associated with reduced CD57 cells. The NK cells can improve immunosurveillance via improving the antibody production by V lymphocytes^32,105^, generation of antitumor cytotoxic T-lymphocytes, and upregulation of MHC molecules^32,105^. On the other hand, Zancope *et al*.^106^ did not detect a significant association between prognosis and NK cell count which might be due to smaller sample size and methodological differences. Also Fraga *et al*.^28^ reported no independent role for CD57 cell density in the tumor with survival although they found significant associations between high CD57+ inflammatory cell density with tumor size and more locoregional metastases; they concluded that a higher density of such cells the peritumoral stroma might lead to an ineffective locoregional antitumoral response^28^. Additionally, Sorbye *et al*.^107^ did not find a significant prognostic role for CD57 cells. Such results might be attributed to the method of CD57 expression evaluation, evaluated cell types, sample types and sizes, tumor types and severities, statistical methods in use, and sample demographics/ethnicities^18,86^. In addition, the location of infiltrating cells might be another reason for controversy as there is difference between epithelial, stromal or peritumoral CD57 positive cells^107^. Moreover, CD57 is expressed also on T lymphocytes which despite their cytotoxic potential are unable to undergo new cell-division^107,108^. Furthermore, tumor-immune system interactions are complicated: sometimes tumors act like subclinical infections evading immune response, and sometimes despite evading immonosurveillance, some of their surface antigens still trigger a progressive (yet inefficient) increase in immune response^89^. The latter might be the case in our study. Also as mentioned above, patients with poorer prognoses might have received stronger treatments, which could confound the results.

The findings of this study indicated a positive role for tumoral TGF-β1 upregulation in survival. Our findings were in contrast to findings of some other studies which failed to show a significant survival role for TGF-β1 in SCCs of head and neck^18,45^ or showed that increased TGF-β1 expression might reduce survival odds^109^. On the other hand, our results were in line with findings of some other studies indicating an association between increased expression of TGF-β1 in the tumor and reduced mortality (and improved survival rate)^107^. The controversy results might be attributed to the complex and dual role of TGF-β1 in tumorigenesis as well differences between pathogenesis of various tumors^7,18,110,111^. Various factors might determine the effect of TGF-β1 including TGF-β1 receptors (normal or diminished), target cell types (normal or tumoral), TGF-β1 dosage, and immune response: while it is mostly tumor suppressor in early tumors, it facilitates tumorigenesis in later stages^7,18,23,36–38,41,44,109–112^; for instance it might enhance^36,41,43^ or inhibit^40,41^ tumor cell invasion. It can facilitate metastasis through increased detachment of cancer cells, tumoral proliferation/invasion, growth stimulation, angiogenesis, MMP induction, or chemoattraction, facilitation of epithelial-to-mesenchymal transition, and increasing invasiveness and motility^8,36–38,40–44,113–118^; while it also can act against the cancer by maintaining the tissue architecture and genomic stability, induction of apoptosis and replicative senescence, attraction of fibroblasts and capsule synthesis, inducing the activity of inhibitors of MMPs, or inhibiting cell proliferation^7,36,38,40–44^. In certain cancers, increased expression of TGF-β1 and its pathway might reduce the production of NK cytokines and CD16 receptor^18,25^, increase CD16 expression^77,80^, downregulate the primary cytotoxicity receptor of NK cells^25,90^, functionally inactivate NK and cytotoxic T cells^88,119,120^, or decrease the suppressor effectiveness of NK cells^121^.

In this study, MED15 overexpression was associated positively with TGF-β1 overexpression. Although TGF-β1 was found to be associated positively with survival, the role of MED15 in survival was negative when the effects of other genes had been controlled for. This result was in contrast with the few other studies finding the opposite. Shaikhibrahim *et al*.^48^ evaluated castration-resistant prostate cancer (CRPC) and reported that MED15 was overexpressed in 76% of distant metastatic and 70% of local-recurrent CRPC versus no expression in benign prostatic tissue; they also found a significant negative association between the overexpression of MED15 and survival^48^. As the only study on head and neck SCC, Shaikhibrahim *et al*.^19^ found MED15 overexpressed in 35% of primary tumors, 30% of lymph node metastases, and 70% of recurrent tumors; they also observed MED15 overexpression to be associated positively with mortality^19^. In this study, MED15 was also positively associated with CD16 and CD57 expressions, which had effects opposite of that of TGF-β1. Based on positive associations with contradicting biomarkers, it seems that its role in survival (if existing) might have been more complicated than a log-linear one.

## Conclusions

Within the limitations of this study, it might be concluded that none of these biomarkers might be suitable for diagnosis of OSCC. Tumoral overexpression of CD16, CD57, and MED15 might predict poorer prognoses, while elevated TGF-β1 levels might be associated with an improved prognosis. The prognosis might be poorer in older patients, cases with higher clinical TNM stages, greater N modes, higher histological grades, and larger/thicker tumors. Cases treated with incisional biopsy might have a poorer prognosis (compared to surgical resection) as well, but this remain inconclusive until more data are collected. Of these variables, only ‘Jacobson clinical TNM staging’ might have a cut-off point for death prediction, i.e., cases with stages above 2 might have a considerably higher risk of mortality.

## Data Availability

The data that support the findings of this study are available from the National Tumor Bank of Iran and the authors, but restrictions apply to the availability of a part of these data, which were used under license for the current study, and so are not publicly available. Data are however available from the authors upon reasonable request and with permission of the National Tumor Bank of Iran.
